# An analysis of functional insole on foot pressure distribution of shape memory material combinations

**DOI:** 10.1186/1757-1146-7-S1-A120

**Published:** 2014-04-08

**Authors:** Seung-Bum Park, Kyung-Deuk Lee, Dae-Woong Kim, Jung-Hyeon Yoo, Kyung-Hun Kim

**Affiliations:** 1 Footwear Biomechanics Team, Footwear Industrial Promotion Center, Busan, Korea

## 

The purpose of this study was to analyze foot pressure distribution of shape memory materials functional insole. Comfort is an important aspect for footwear and insole. Footwear and insole comfort has an influence on injury [1,2]. The development of new materials is considered as the important point for manufacturing functional insole [3,4].

Ten healthy male (mean height: 174.7±4.0 cm, mean body mass: 71.0±8.0 kg, mean age 23.9±0.3 yrs.) were participated in this study. All subjects were free of lower extremity pain, history of serious injuries or operative treatment or subjective symptoms interfering with walking. Each subject's foot was pre-screened by Podoscopy (Alfoots, Korea) to see if they had any foot abnormalities.

**Figure 1:**
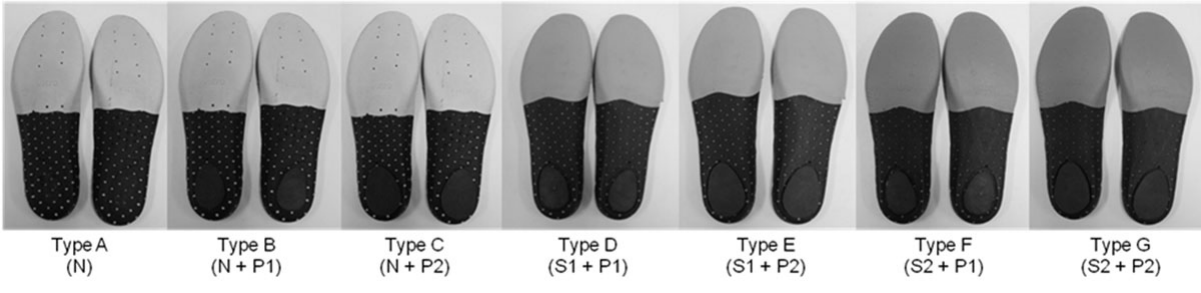
Tested seven types insoles (L-R): Type A ~ G. N: normal material, S1: low hardness shape memory material, S2: high hardness shape memory material, P1: low hardness Poron^®^ material, P2: high hardness Poron^®^ material**.**

The subjects were required to normal walking (4.2km/h) for treadmill. Each subjects was seven different insole type (A ~G type, figure 1) during walking. The PEDAR^®^-X insole system (Novel GmbH, Germany) was used to measure the foot pressure and force. Pressure distribution data (peak pressure, maximum mean pressure) was collected with pressure device at a sampling rate of 100Hz. The feet were divided into six regions: foot (Total), lateral forefoot (M1), medial forefoot (M2), midfoot (M3), lateral rearfoot (M4), and medial rearfoot (M5).

**Figure 2:**
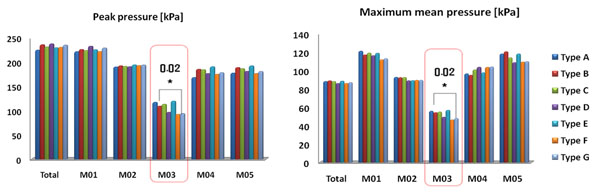
Comparison of foot pressure of the seven types insoles.

Comparison of foot pressure is show in figure 2. In the midfoot (M3) area, a significant different was found between insoles in peak pressure and maximum mean pressure. The type F and G insoles decreased the peak pressure and maximum mean pressure.
